# COVID-19 vaccine uptake, barriers and associated factors among healthcare workers in Malawi

**DOI:** 10.4102/jphia.v16i1.676

**Published:** 2025-02-14

**Authors:** Clara Sambani, Tonny Muwonge, Liness Chinyamunyamu, Amon Dembo, Mosoka P. Fallah, Victor Chikwapulo, Mavuto Thomas, Charity Gondwe, Regina Mankhamba, Leah Mbabazi, Suzan Nakasendwa, Rodgers R. Ayebare, Collins Mitambo, Matthew Kagoli, Dzinkambani Kambalame, Clement Seven, Tadala Mwale, Edna Mandala, Abigail Kazembe, McWilliam Kalua, Senga Sembuche, Elizabeth Gonese, Tamrat Shaweno, Nebiyu Dereje, Evelyn C. Banda, Tajudeen Raji, Mitch Matoga

**Affiliations:** 1 Public Health Institute of Malawi, Lilongwe, Malawi; 2 Infectious Diseases Institute, Makerere University, Kampala, Uganda; 3 Ministry of Health, Lilongwe, Malawi; 4 Kamuzu Central Hospital, Lilongwe, Malawi; 5 Africa Centres for Disease Control and Prevention, Liberia; 6 World Health Organization, Lilongwe, Malawi; 7 Kamuzu University of Health Sciences, Blantyre, Malawi; 8 Malawi Ministry of Health Extended Program of Immunization, Lilongwe, Malawi; 9 Africa Centers for Disease Control and Prevention, United Republic of Tanzania; 10 Africa Centers for Disease Control and Prevention, Zimbabwe; 11 Africa Centers for Disease Control and Prevention, Addis Ababa, Ethiopia; 12 University of North Carolina Project, Charlotte, United States of America

**Keywords:** COVID-19, healthcare workers, vaccine, uptake, booster, Malawi

## Abstract

**Background:**

The COVID-19 pandemic led to an urgent need for a global vaccine. Despite being a priority group, the vaccine uptake among healthcare workers (HCWs) remains low.

**Aim:**

This article assessed the COVID-19 vaccine uptake and its associated barriers in Malawi.

**Setting:**

A cross-sectional study was conducted among HCWs in Malawi’s Blantyre, Lilongwe and Mzuzu districts from 11 April 2023 to 14 April 2023.

**Methods:**

Proportionally distributed among various cadres, 200 participants were randomly selected. A structured questionnaire was administered, assessing HCWs’ uptake, willingness, attitudes and barriers to COVID-19 vaccines. Data were managed in REDCap and analysed using STATA version 14. Frequencies and percentages were computed. Variables with *p*-value ≤ 0.25 were included in the multivariable modified passion model.

**Results:**

A total of 175 (88%) participants received a single-dose vaccine, while only 11.5% received a booster. Vaccine uptake was associated with age groups 25–34 years (aPR = 2.35, 95% CI: 1.21, 4.60) and 35–44 years (aPR = 2.30, 95% CI: 1.18, 4.50), being a nurse/midwife (aPR = 0.86, CI: 0.74, 0.99) and laboratory personnel (aPR = 0.86, 95% CI: 0.74, 0.99). Unvaccinated HCWs were concerned about vaccine side effects and lacked trust in the development process.

**Conclusion:**

Issues of vaccine safety, side effects and lack of trust in the vaccine should be addressed. Promoting awareness of vaccine development and benefits, targeting all age groups and cadres, is required among HCWs.

**Contribution:**

The findings can be utilised to develop specific interventions on age and cadre to promote vaccine acceptance among HCWs, in countries with similar contextual settings to Malawi.

## Introduction

The coronavirus disease 2019 (COVID-19) vaccination campaigns in Africa focussed on priority groups, including healthcare workers (HCWs), teachers and older people.^1,2^ By the end of 2021, the global distribution of the COVID-19 vaccines was more than 12 billion doses.^3^ Malawi received 2 425 790 vaccine doses by October 2021.^4^ There was an evident and marked inequality in COVID-19 vaccine distribution.^3^ The need for global vaccine equity eventually led to an increased supply of vaccines across Africa, including Malawi.^2^ In the first phase of vaccination, the targeted population groups in Malawi were those at high risk of mortality from COVID-19 and the HCWs. From May 2021, the vaccine access was extended to include all people aged above 18 years, including pregnant women and marginalised people such as prisoners and refugees in the Malawian population.^4^ As of 22 November 2023, Africans had been vaccinated with approximately 1137.4 million doses, with only 31.1% being fully vaccinated. Within the same period, approximately 30.2% of Malawians inclusive of HCWs had been fully vaccinated, and only 8.1% had received a booster dose.^5^

Despite the increased access to efficacious COVID-19 vaccines for Africa, many people, including HCWs, remain unvaccinated because of prevailing misconceptions about the vaccines.^6^ The low uptake in African countries is associated with perceptions about vaccines. People have some vaccine hesitancy as they refuse or delay vaccinations and remain undecided about the use of vaccines.^6,7^ As expected, the low uptake and associated perceptions have also affected the HCWs. A Malawian study showed that 17.5% of HCWs have not yet received a single dose of the vaccine.^8^ This study was carried out in the very early days of COVID-19 vaccine availability in Malawi and did not include the northern region. Other surveys and in-depth interviews with HCWs have highlighted reasons for low uptake of COVID-19 vaccine, including distrust in the vaccine because of rushed development, fear of uncertainty in the use of the vaccine and its associated side effects, social pressure and the media effect because there was a lot of misinformation and a lack of clarity.^9,10,11^ A recent review found that COVID-19 vaccine acceptance among African HCWs was influenced by factors such as vaccine availability, misinformation and cultural beliefs.^12,13^ In South Africa, for example, initial vaccine uptake among HCWs was high, but there is resistance to receiving booster doses because of safety concerns, a lack of trust in government information and the perception of low infection risk after the initial vaccination.^13^

Health workers are usually the trusted source of information for the community^14^ and they play a crucial role in the actual provision of care, such as vaccination. This entails that HCWs’ hesitancy to receive COVID-19 vaccination could not only affect their health but also their patient’s health, their communities and the health system, as they may be less likely to advocate for the vaccine.^14,15^ Nevertheless, HCWs should always be protected through the vaccines as they are also at a higher risk of getting infected with COVID-19. Considering the risks and negative impact of having unvaccinated HCWs, this study was crucial in identifying and addressing persistent barriers to COVID-19 vaccine uptake among health workers which may have persisted even after more than 2 years of COVID-19 vaccine administration in Malawi. The authors assessed the COVID-19 vaccine uptake among HCWs in Malawi and the barriers associated with this uptake.

## Research methods and design

### Study design and population

This was a descriptive, cross-sectional study conducted at various facilities. The study was conducted in Blantyre, Lilongwe and Mzuzu districts of Malawi, chosen for their high COVID-19 burden and earlier vaccine access. During the pandemic, most of the COVID-19 cases were referred to these three districts. Participants were HCWs from three tertiary and two primary healthcare facilities within these districts. Selection focussed on facilities with high patient volumes (central hospitals) or high COVID-19 caseloads.

### Sampling strategy

A sample of 200 HCWs was recruited. The sampling aimed for representation across cadres, with pre-calculated sample size and proportional distribution among the cadres: 60 clinicians (doctors, officers and assistants), 40 nursing and midwifery staff, 20 pharmacy personnel (pharmacists, technicians and assistants), 40 laboratory personnel (scientists, technicians and assistants), 20 community health workers (public health experts, environmental health workers and health promotion officers) and 20 other HCWs (radiographers and anaesthetists). On the day of the interview, a list of HCWs who were on duty was obtained from the selected health facilities and served as the sampling frame. The authors then randomly sampled the participants according to their cadres to ensure that all cadres were represented in the sample. Two-thirds of participants were recruited from central hospitals, reflecting their larger patient populations. Healthcare workers who provided written informed consent were included.

### Data collection

Data were collected from 11 April 2023 to 14 April 2023, using a structured questionnaire translated into Chichewa and Tumbuka. Research assistants with adequate research experience were recruited and trained on the protocol and data collection procedures including practising the use of mobile devices. During data collection, the research assistants administered the questionnaire, which assessed HCW vaccination status, attitudes and barriers. The questionnaire covered demographics including age, sex and cadre; vaccination details including whether they thought the vaccine would protect people from infectious diseases or reduce the risk of dying from COVID-19 and whether they had been vaccinated; a list of possible reasons for non-vaccination, and vaccine importance and advocacy perceptions on whether they could recommend the vaccine to eligible people. Data were collected electronically using REDCap on tablets that were password-protected to ensure that only the study staff had access to the information. On a daily basis, all tablets were handed over to the supervisor to maximise the safety of the data.

### Data analysis

Nine research assistants were trained and entered data in real-time to ensure completeness and accuracy. Data quality checks were conducted periodically in REDCap. Data were exported to STATA 14 for cleaning and analysis.

Descriptive statistics were used to describe demographics (means or medians for continuous data, proportions or frequencies for categorical data). Proportions of uptake, barriers and enhancers of vaccination were calculated and compared using Chi-square tests. The binary analysis identified candidate variables for a modified Poisson regression model. Variables with *p*-values < 0.25 in the binary analysis were included in the final model.

The modified Poisson regression model identified risk factors associated with vaccine uptake at a 95% confidence interval (CI). Adjusted prevalence ratios (aPR) with 95% CIs were used to assess associations between explanatory and dependent variables. A *p*-value < 0.05 was considered statistically significant.

### Ethical considerations

Ethical approval was obtained from the College of Medicine Research Ethics Committee (NHSRC #01/23/3945). Written informed consent was obtained from all participants. Confidentiality and privacy were ensured. Participants could withdraw at any point. Only the study team had access to the collected data.

## Results

### Demographic characteristics and vaccination status of the participants

Out of the 200 HCWs interviewed in the survey, more than half (57.0%, *n* = 114) were within 25–34 years and 53.0% (*n* = 106) were male. Most (26.0%, *n* = 52) of the HCWs were clinicians seconded by nurses and midwives (23.0%, *n* = 46). Eighty-eight per cent (*n* = 175) of the HCWs received a single dose of COVID-19 vaccine, while only 11.5% received a booster. Regarding the preferences of vaccination centres among HCWs, hospitals and health centres were the most preferred choices (*n* = 170, 85.0% and *n* = 95, 47.5%, respectively) (Table 1).

**TABLE 1:** Demographic characteristics and vaccination status of the participants *N* = 200.

Characteristics	Frequency			
	Median	IQR	*n*	%
**Age in years**	28	32–38	-	-
21–24	-	-	13	6.5
25–34	-	-	114	57.0
35–44	-	-	50	25.0
45–54	-	-	19	9.5
55 and above	-	-	4	2.0
**Gender (*N* = 200)**				
Female	-	-	94	47.0
Male	-	-	106	53.0
**Cadre (*N* = 200)**				
Clinicians†	-	-	52	26.0
Nursing and midwifery	-	-	46	23.0
Pharmaceutical personnel	-	-	17	8.5
Laboratory health worker	-	-	40	20.0
Community support and public health worker	-	-	18	9.0
Other health workers‡	-	-	27	13.5
**Health facility (*N* = 200)**				
Area 25 Health Centre	-	-	16	8.0
Kamuzu Central Hospital	-	-	52	26.0
Mapale Health Centre	-	-	12	6.0
Mzuzu Central Hospital	-	-	54	27.0
Queen Elizabeth Central Hospital	-	-	66	33.0
**Vaccination status (*N* = 198)**				
Booster dose	-	-	22	11.0
Full vaccination	-	-	133	66.5
Partial vaccination	-	-	20	10.0
Unvaccinated	-	-	23	11.5
**Preference for vaccination centre (multiple responses)**				
Hospital	-	-	170	85.0
Health centre/clinic	-	-	95	47.5
Community centre, meeting hall or local shop	-	-	53	26.5
Pharmacy	-	-	14	7.0
Do not want to vaccinate	-	-	12	6.0
Somewhere else (Church, home, and outreaches)	-	-	8	4.0

IQR, interquartile range.

† , Clinicians include medical doctors, clinical officers and medical assistants;

‡ , Other health workers include radiographers and anaesthetic officers.

### Barriers affecting the uptake of the vaccine among healthcare workers

More than half (53.3%, *n* = 24) of the HCWs who were not fully vaccinated or boosted indicated that they were concerned about the safety and side effects of the vaccine. Some HCWs highlighted other reasons, including a lack of interest, fear, pregnancy and having a strong immunity. Among those who would not want to take any vaccine or booster, 36% (*n* = 9) of the HCWs expressed concerns that the development and authorisation of the vaccine were rushed and it may not have been thoroughly tested, and some (32%, *n* = 8) were concerned about severe side effects like blood clots, neurological disorders and effects on motherhood (Table 2).

**TABLE 2:** Barriers affecting the uptake of COVID-19 vaccine among healthcare workers.

Reasons for not receiving full vaccination or booster	Frequency†		Frequency‡	
	*n*	%	*n*	%
**Reasons why HCWs were not fully vaccinated or boosted (multiple responses)**				
I am concerned about the safety and side effects of the vaccines	24	53.3	-	-
Other reasons (a lack of interest, fear, pregnancy and strong immunity)	21	46.7	-	-
I am waiting to see how the vaccine affects other people that I know	7	15.6	-	-
I do not have time to get vaccinated	3	6.7	-	-
I do not wish to respond	2	4.4	-	-
Waiting for the window of eligibility to open	2	4.4	-	
I do not want to miss work	1	2.2	-	-
I do not have transportation to get to the vaccination site	1	2.2	-	-
I do not know where to go to get a vaccine	1	2.2	-	-
**Reasons why HCWs would not want to receive an approved COVID-19 vaccine and/or booster (multiple responses)**				
I feel the development and/or authorisation of the vaccine was rushed and it may not be thoroughly tested	-	-	9	36.0
I am concerned about serious side effects like blood clots, neurological disorders and effects on motherhood	-	-	8	32.0
Vaccines are against my religious beliefs	-	-	5	20.0
I already had COVID-19 and am not worried about being infected again	-	-	4	16.0
I do not feel I am at risk of getting very sick or dying from the virus	-	-	4	16.0
Other concerns about conspiracies they might have heard about include micro-chips, sterilisation, tracking, etc.	-	-	3	12.0
I do not feel I am at risk of catching the virus	-	-	2	8.0
Someone in my family or community does not want me to get the vaccine	-	-	1	4.0
The vaccine available in my country will not protect me	-	-	1	4.0
Vaccines can give you the disease they are designed to protect you against	-	-	1	4.0
I do not yet know enough about the vaccine to decide	-	-	1	4.0

HCW, healthcare worker; COVID-19, coronavirus disease 2019.

† , *n* = 45 for each response;

‡ , *n* = 25 for each response.

**FIGURE 1:**
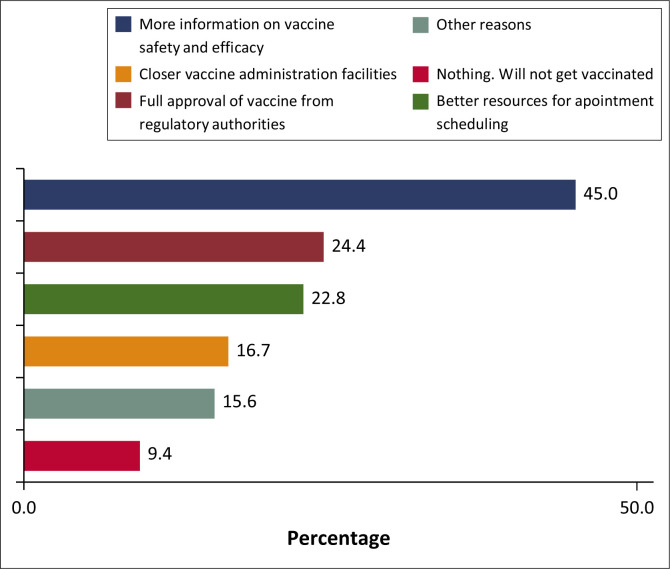
The most probable solution to help healthcare workers decide whether to get vaccinated or boosted.

Some (45%) of the HCWs believed that offering them more information about COVID-19 vaccine safety and efficacy would help HCWs’ decision to get vaccinated or boosted. Others (24.4%) indicated that their choice would be influenced by having full approval of vaccines from regulatory authorities (Figure 1).

Most HCWs (80.0%, *n* = 160) strongly agreed that vaccinating against infectious diseases such as measles and tuberculosis reduces the risk of a person getting sick or dying. Although lower, most (57.5%, *n* = 115) also strongly agreed that vaccination against COVID-19 minimises the risk of a person getting ill or dying (Table 3).

**TABLE 3:** Comparison of perceived effectiveness of COVID-19 vaccine against other infectious diseases vaccines.

Number	HCWs’ level of perception‡	COVID-19		Other infectious diseases†	
		*n*	%	*n*	%
1	Strongly disagree	12	6.0	19	9.5
2	Disagree somewhat	4	2.0	0	0.0
3	Neither agree nor disagree	7	3.5	1	0.5
4	Agree somewhat	62	31.0	18	9.0
5	Strongly agree	115	57.5	160	80.0
6	Did not wish to respond	0	0.0	2	1.0

HCW, healthcare worker; COVID-19, coronavirus disease 2019.

† , Other infectious diseases, that is, childhood immunisations, for example, measles and tuberculosis;

‡ , HCWs’ level of perception on whether being vaccinated against COVID-19 or infectious diseases reduces the risk of a person getting sick or dying.

### Factors associated with uptake of COVID-19 vaccine

The COVID-19 vaccine uptake was associated with age groups ranging between 25–34 years (aPR = 2.35, 95% CI [1.21, 4.60]), 35–44 years (aPR = 2.30, 95% CI [1.18, 4.50]), being a nurse and midwife (aPR = 0.86, 95% CI [0.74, 0.99]) and also being a laboratory health worker (aPR = 0.86, 95% CI: [0.74, 0.99]). Thus, those between 25 years and 44 years were about 2.3 times more likely to get a vaccine than HCWs aged below 25 years. Those in nursing, midwifery and laboratory were 16% less likely to get vaccinated than clinicians (aPR = 0.86; 95% CI: 0.74–0.99 for both). The healthcare structure was not associated with vaccine uptake (Table 4).

**TABLE 4:** Modified Poisson regression model for the uptake of COVID-19 vaccine among vaccinated and unvaccinated healthcare workers.

Characteristic	Crude		Adjusted		Adjusted *p*-value
	PR	95% CI	PR	95% CI	
**Age in years**					
21–24	Ref	Ref	Ref	Ref	-
25–34	2.37	1.19, 4.73	2.35	1.21, 4.60*	0.012
35–44	2.34	1.17, 4.69	2.30	1.18, 4.50*	0.015
45–54	-	-	-	-	-
55–57	-	-	-	-	-
**Gender**					
Female	Ref	Ref	Ref	Ref	-
Male	1.03	0.93, 1.14	0.96	0.86, 1.07	0.482
**Cadre**					
Clinicians	Ref	Ref	Ref	Ref	-
Nursing and midwifery	0.84	0.72, 0.96*	0.86	0.74, 0.99*	0.043
Pharmaceutical personnel	0.85	0.66, 1.08	0.83	0.65, 1.06	0.126
Laboratory health worker	0.83	0.71, 0.98*	0.86	0.74, 0.99*	0.043
Community support and public health worker	0.98	0.87, 1.11	0.95	0.82, 1.11	0.559
Other health workers	-	-	-	-	-
**Healthcare structure**					
Tertiary	-	-	Ref	Ref	-
Primary	0.96	0.82, 1.13	0.96	0.82, 1.11	0.563

PR, prevalence ratio; aPR, adjusted prevalence ratio; Ref, reference category; CI, confidence intervals; COVID-19, coronavirus disease 2019.

* , Statistically significant at *p* < 0.05.

## Discussion

In this population of HCWs from three large cities in Malawi, about two-thirds of HCWs were fully vaccinated for COVID-19. Most of those not fully vaccinated were concerned about side effects, the overall safety of the vaccines, and the rush in the development process of the vaccines. Older HCWs and clinicians were more likely to get vaccinated.

The majority (88.0%) of the HCWs had received at least one dose of the COVID-19 vaccine. This uptake was higher compared to other studies in Ethiopia and Malawi, which found that 62.1% and 82.5% had received at least one dose, respectively.^8,16^ The policy and emphasis that HCWs were a target or priority group for vaccination could have increased the uptake of the initial doses. However, despite the high proportion of single-dose vaccination, fewer (66.0%) HCWs were fully vaccinated. In addition, uptake (11.0%) of the booster dose was lower than the 56.0% booster uptake for a study in South Africa.^13^ Our HCWs may not understand the importance and effectiveness of receiving the COVID-19 booster dose or need more information on the importance of getting a booster.^17,18^ There is a need to ensure that HCWs are continuously trained and are made aware of the benefits of booster doses. Some of the reasons for the hesitancy include distrust in the vaccine because of its rushed development, fear of uncertainty about its use and its associated side effects, social pressure and the media’s effect because there was a lot of misinformation and non-clarity.^9,10,11^ Continuous professional development and other messaging within the facilities would be useful and can build more confidence in booster doses among the HCWs.

It has been shown that the different HCW cadres differ in their uptake of COVID-19. In our study, many (94.0%) clinicians, anaesthetists and radiographers had at least one vaccine, while nurses and laboratory staff were the least (80.0%) to have received a COVID-19 vaccine. In a different study in Malawi, higher-rated cadres showed a higher vaccination uptake rate (87.6%) than lower cadres (79.9%).^8^ Higher acceptance and uptake for COVID-19 vaccination among physicians was also noted in Algeria and New York.^19,20^ Similarly, a secondary data analysis from 23 countries also showed that physicians dominated vaccination than other cadres.^21^ In the Malawian health system, clinicians are seen to be more senior in the chain of command compared to other cadres, and this could explain why more clinicians in our study were vaccinated compared to other different cadres. Even socially, the higher-rated cadres are often viewed in the community as role models and this could influence their behaviour desiring to adhere to socially acceptable norms such as getting vaccinated.^9^ Additionally, it is common that the higher-rated HCW cadres have more access to information, training and networking opportunities with external colleagues and experts; hence, they would have valuable insights that influence their decision to take the vaccine.

Among the reasons for not being vaccinated or boosted, 36.0% of HCWs felt the development and authorisation of the vaccine was rushed, and it may not have been thoroughly tested and validated. Generally, 53.3% of the health workers who were not fully vaccinated or boosted were equally concerned with the safety and side effects of the vaccines. This notion was also reflected by the perception that vaccines for other infectious diseases are more potent than COVID-19. In another study, 67.0% of HCWs preferred the influenza vaccine compared to 35.0% who preferred the COVID-19 vaccine, and the COVID-19 vaccine was reported to have more side effects than the influenza vaccine.^22^ These concerns might indeed directly affect HCWs’ decision-making on whether to get the vaccine or not. Some HCWs (15.6%) in our study were still waiting to observe how those who had received the vaccine would fare before they could decide to get the jab. A scoping review for Africa also found similar findings.^7^

The HCWs believed that more information was needed to help them make decisions about getting COVID-19 vaccines. Similarly, a systematic review on COVID-19 vaccine acceptance among HCWs in Africa also found that HCWs lacked information on the processes used to produce the vaccine hastily, which made them hesitant to receive the vaccine.^12^ On the other hand, 57.5% of the HCWs strongly agreed that vaccination against COVID-19 reduces one’s risk of getting sick or dying. In the United Kingdom, Burrowes et al. found that HCWs generally believed vaccination would protect them and their families from COVID-19 infection.^23^

Older HCWs were twice as likely to be vaccinated as younger HCWs. Other studies also found that those who are 30 years and above were more likely to get vaccinated than young ones. Because older people are more at risk of severe COVID-19,^24,25,26^ this may explain why the older HCWs were more likely to be vaccinated. Older HCWs in our study might have been worried about getting severe disease if infected with COVID-19.

### Broader public health implications

The findings from this study provide important insights that can inform vaccine rollout strategies and public health communication, particularly in resource-limited settings like Malawi. Several key implications can be drawn to address the low booster dose uptake and the vaccine hesitancy observed among certain HCW groups:

- Targeted communication and training for HCWs: Our study highlights that hesitancy regarding COVID-19 vaccines and boosters is often driven by concerns over vaccine safety, side effects and the speed of vaccine development. To address this, there is a need for continuous professional development and training that emphasises the rigorous processes behind vaccine development and approval. This training can help build confidence in the vaccine’s safety, particularly among HCWs who are hesitant or have expressed doubts. Tailored communication strategies that address these specific concerns, possibly through targeted messaging within healthcare facilities, can improve uptake.
- Boosting confidence in booster doses: With only 11.0% of HCWs having received the booster dose, there is a clear gap in understanding its importance. Efforts should focus on educating HCWs about the role of booster doses in maintaining immunity, especially as the COVID-19 pandemic evolves. Facility-based information sessions, as well as peer-led initiatives, could encourage HCWs to trust in the effectiveness of booster doses, thereby improving coverage.
- Addressing misinformation and social pressures: Many HCWs in the study cited concerns about the vaccine’s rushed development and uncertainty about its side effects, compounded by misinformation. Public health authorities should implement strategies to counteract misinformation through transparent communication and engagement. This could involve partnering with respected figures in the healthcare system, such as clinicians and senior staff who have higher vaccine acceptance rates, to serve as champions of the vaccine and boost trust among other cadres, particularly nurses and laboratory staff who had lower uptake rates.
- Tailoring approaches based on cadre-specific uptake: Our findings indicate variation in vaccine uptake across different HCW cadres. For example, clinicians, anaesthetists and radiographers had higher vaccination rates compared to nurses and laboratory staff. Vaccine rollout strategies could benefit from cadre-specific approaches, where higher-rated cadres like clinicians lead by example and assist in promoting vaccination among their colleagues in other categories. This peer-to-peer encouragement could help address disparities in vaccine acceptance within healthcare settings.

Future pandemic preparedness: The lessons from this study can also be applied to future public health crises. Addressing the specific concerns of HCWs will be crucial in improving response efforts for potential future pandemics. Proactive measures, such as building trust through early education and clear, transparent communication, should be prioritised to mitigate vaccine hesitancy from the outset of any public health campaign.

### Limitations

The participants were purposively sampled and interviewed at their workplace. There could be some possible social desirability bias because it was difficult to verify some of the information given, such as vaccination status, and most HCWs who accepted to be interviewed had likely been vaccinated, thereby overestimating the uptake of COVID-19 vaccines among HCWs, which may limit the generalisability of the results. However, the authors ensured that various cadres should be involved to diversify the responses. Research assistants stressed that the responses would be confidential and only available to the study team to encourage honest reporting.

Additionally, this study relied on self-reported data, which may be subject to recall bias, particularly for booster dose uptake. The cross-sectional design also limits our ability to establish causal relationships between factors like age and professional cadre with vaccine uptake. Future longitudinal studies could better capture these dynamics.

The study did not assess how many health workers suffered from COVID-19, how many died or HCWs’ underlying chronic conditions, which might be some of the barriers or enablers to vaccination. Moreover, the lack of qualitative insights limited our ability to explore in-depth reasons behind vaccine hesitancy, particularly for the booster dose. Future research should consider incorporating qualitative methods to better understand these aspects. However, the authors managed to exhaust many other possible reasons influencing the uptake of COVID-19 vaccine.

Finally, the study’s findings may have limited generalisability beyond the Malawian healthcare system. Cultural, economic and structural factors specific to Malawi may influence HCWs’ vaccine uptake differently compared to those in other countries. Therefore, caution should be exercised when applying these results to other contexts different from the Malawian setting.

## Conclusion

The uptake of COVID-19 vaccines among HCWs in three big cities of Malawi was sub-optimal. Barriers to the uptake of COVID-19 vaccines can be addressed by sensitising HCWs through comprehensive education on the COVID-19 vaccine. Sensitising HCWs to COVID-19 vaccines and other available vaccines would not only result in increased vaccine uptake among HCWs, but would consequently increase vaccine uptake in communities. Most HCWs who were not yet vaccinated were concerned about vaccine safety, including their side effects. Other HCWs had no interest in COVID-19 vaccination, had a fear of the vaccine and had a claim of strong immunity against COVID-19. Although many HCWs had strong confidence in COVID-19 vaccines, they believe that full approval from regulatory authorities will help them decide to get vaccinated or boosted. This gap necessitates continued sensitisation of HCWs on vaccines, their development, approval process, effectiveness and safety profiles.

To promote vaccine uptake, HCWs should be prioritised and offered enough information about emerging infections, their vaccines, including the side effects and their management. This will help address health workers’ concerns about vaccination, thereby enabling them to recommend the COVID-19 vaccine and any other concerned vaccine to patients and the community. Further country-wide studies are needed to identify effective communication models for HCWs and compare vaccine hesitancy levels between HCWs with high burden of COVID-19 disease and those with a low burden.
